# Autophagy and Apoptosis Are Differentially Induced in Neurons and Astrocytes Treated with an *In Vitro* Mimic of the Ischemic Penumbra

**DOI:** 10.1371/journal.pone.0051469

**Published:** 2012-12-12

**Authors:** Matthew E. Pamenter, Guy A. Perkins, Anelah K. McGinness, Xiang Q. Gu, Mark H. Ellisman, Gabriel G. Haddad

**Affiliations:** 1 Department of Pediatrics (Division of Respiratory Medicine), University of California San Diego, La Jolla, California, United States of America; 2 National Center for Microscopy and Imaging Research, University of California San Diego, La Jolla, California, United States of America; 3 Center for Research in Biological Systems, University of California San Diego, La Jolla, California, United States of America; 4 Department of Neuroscience, University of California San Diego, La Jolla, California, United States of America; 5 The Rady Children’s Hospital-San Diego, San Diego, California, United States of America; Massachusetts General Hospital/Harvard Medical School, United States of America

## Abstract

The development of clinical stroke therapies remains elusive. The neuroprotective efficacies of thousands of molecules and compounds have not yet been determined; however, screening large volumes of potential targets *in vivo* is severely rate limiting. High throughput screens (HTS) may be used to discover promising candidates, but this approach has been hindered by the lack of a simple *in vitro* model of the ischemic penumbra, a clinically relevant region of stroke-afflicted brain. Recently, our laboratory developed such a mimic (ischemic solution: IS) suitable for HTS, but the etiology of stress pathways activated by this model are poorly understood. The aim of the present study was to determine if the cell death phenotype induced by IS accurately mimics the *in vivo* penumbra and thus whether our model system is suitable for use in HTS. We treated cultured neuron and astrocyte cell lines with IS for up to 48 hrs and examined cellular energy state ([ATP]), cell and organelle morphology, and gene and molecular profiles related to stress pathways. We found that IS-treated cells exhibited a phenotype of mixed apoptosis/autophagy characteristic of the *in vivo* penumbra, including: (1) short-term elevation of [ATP] followed by progressive ATP depletion and Poly ADP Ribose Polymerase cleavage, (2) increased vacuole number in the cytoplasm, (3) mitochondrial rupture, decreased mitochondrial and cristae density, release of cytochrome C and apoptosis inducing factor, (4) chromatin condensation, nuclear lamin A and DNA cleavage, fragmentation of the nuclear envelope, and (5) altered expression of mRNA and proteins consistent with autophagy and apoptosis. We conclude that our *in vitro* model of the ischemic penumbra induces autophagy and apoptosis in cultured neuron and astrocyte cell lines and that this mimic solution is suitable for use in HTS to elucidate neuroprotective candidates against ischemic penumbral cell death.

## Introduction

Cells in the infarct core die within minutes of stroke onset, whereas in the surrounding region (the penumbra), death spreads slowly for hours to days post-insult [1], [2]. Unlike the infarct core, the relatively slow propagation of cell death in the penumbra makes this region an attractive target for clinical rescue, particularly as the majority of stroke-related morbidity and mortality is attributable to progressive expansion of the infarct core into the penumbra [3]. The mechanism(s) of cell death in this region are poorly understood, but *in vivo* experiments indicate that both apoptosis and autophagy are activated [3], [4], [5], [6], [7], [8]. These responses are likely initiated by alterations of the local perfusate following the release of cytoplasmic contents from ruptured core cells [9]. Indeed loss of membrane integrity is a commonly-shared hallmark of cell-death pathways [8] and facilitates the release of pro-apoptotic and -immunogenic signals, ions, and other debris from dying cells, which accumulate in the local perfusate and initiate stress pathway responses in adjacent cells [9], [10], [11]. In ischemic pathology these effects are compounded by reduced cerebral blood flow, which slows the removal of extruded signaling molecules, ions, and metabolically-derived lactate and CO_2_; thereby enhancing cytotoxic signal accumulation, ionic imbalance, and acidification in the penumbral milieu [11], [12], [13]. Thus the penumbra is exquisitely vulnerable to deleterious signals released from ruptured cells in the nearby infarct core; and elucidating the pathways that underlie ischemic pathology in the penumbra and the spread of cell death and inflammation following stroke are of pressing clinical interest.

Despite extensive research efforts and many clinical trials, neuroprotective agents for ischemia-challenged brain cells remain elusive. Many unexplored molecules and compounds exist that may provide neuroprotection, but screening the efficacy of these on an *in vivo* scale is time-consuming and relatively ineffective. High throughput screens (HTS) in cell lines offer a rapid means to examine large libraries of potentially neuroprotective compounds, but such examinations require a comprehensive mimic of the targeted milieu: the penumbra. While penumbral cell death mechanisms have only recently begun to be elucidated *in vivo* [6], [14], most *in vitro* examinations to date have relied on simple models of acute ischemia such as oxygen-glucose deprivation, chemical ischemia (i.e. cyanide-induced), or N_2_-gassing. These mimic some of the regional effects of acute occlusion (i.e. reduced O_2_%, metabolic inhibition), but not the local effects of cellular rupture that are key to spreading death in the penumbra [15], [16]. To address this need, ‘ischemic solution’ (IS) mimics the key ionic, pH, O_2_%, glucose, and neurotransmitter changes previously discovered in the ischemic penumbra *in vivo* [11], [17]. Preliminary investigations indicate that IS induces deleterious reactive oxygen species generation and up-regulation of innate immune pathways in primary neurons and cell lines, similar to changes observed in the ischemic penumbra *in vivo* [18], [19], [20], [21]. Nonetheless, the cell death and stress pathways activated by this penumbral mimic model remain poorly understood.

The aims of the present study were 1) to examine the effect of IS on cell lines suitable for HTS and 2) to determine the morphological and molecular phenotypes, and thereby the stress pathways, induced by IS-treatment, for comparison to the penumbra *in vivo*. We treated neuronal and astrocytic cell lines with IS, the ATP-competitive non-specific kinase inhibitor staurosporine (STS) [22], which induces apoptosis by activating caspase-3 [23], or the mitochondrial ATP-synthase antagonist oligomycin A to rapidly deplete cellular energy stores and induce autophagy [24]. To assay stress pathway activation we utilized transmission electron microscopy (TEM) to examine whole cell and organelle phenotypes, and also examined [ATP] and gene and protein changes related to autophagy and apoptosis, to provide insight into the molecular signature that contributes to the observed cellular phenotypes.

## Materials and Methods

### Cell Cultures

HT22 mouse hippocampal neurons (a gift from Dr. Pam Maher, Salk Institute, La Jolla, CA [25]) and C8D1A mouse type-I astrocytes (ATCC, Manassas, VA) were cultured in Dulbecco’s Modified Eagle Medium (DMEM, ATCC) supplemented with 10% bovine calf serum (Hyclone, Santa Clara, CA) and 100 U/ml penicillin/streptomycin (Invitrogen, Carlsbad, CA) and grown at 37°C in a 5% CO_2_ incubator. Cells were grown for 5–8 passages and split when they reached 60–80% confluence. For experiments, cells were seeded into 96- or 384-well microplates (Corning, Lowell, MA), glass-bottom 35 mm culture dishes (MatTek, Ashland, MA), or cell culture flasks (Corning) at a density such that when grown overnight they reached ∼70% confluence for experimentation. Samples were treated as specified in the *experimental design* section (below). To reduce sheer stress, cells seeded into multi-well microplates were gently washed with a TECAN PW96/384 Washer (TECAN, San Jose, CA) and then examined visually to ensure cells had not been washed away.

### Experimental Design

Samples were treated for up to 48 hrs (as indicated) in four primary treatment categories: (1) cell death-negative control (“treatment media”, comprised of DMEM/F12 media (Invitrogen) supplemented with 1% bovine calf serum and 1% Pen/Strep), gassed with 21% O_2_, 5% CO_2_, balance N_2_, (2) an ischemic penumbral perfusate mimic (IS, in mM: K^+^64, Na^+^51, Cl^−^ 77.5, Ca^2+^0.13, Mg^2+^1.5, glucose 3.0, glutamate 0.1, [315 mOsM, pH 6.5, 1.5% O_2_, 15% CO_2_, balance N_2_]) [11], [17], [20], (3) an apoptosis-positive control (treatment media containing the pro-apoptotic agent STS (2.5 µM)), and (4) an autophagy-positive control (treatment media containing Oligomycin A (10 µM)). Many stages of apoptosis are ATP-dependent [26], therefore we chose to examine cellular viability and cell death pathway activation in the first 24–48 hours of treatment since in ATP-luciferase experiments cellular [ATP] was wholly depleted in all non-control experimental groups and cell types following 24–48 hours of treatment. Therefore we reasoned that markers indicative of the death pathway fate choice induced in each cell type would be most apparent within this time period. All treatments were run simultaneously and in parallel for each assay. Following treatment, samples were assayed as indicated below. Experimental concentrations of STS and Oligomycin A used in this study are similar to other *in vitro* examinations using these brain cell lines [27], [28], were selected to match previous examinations in our laboratory [21], and were initially chosen based on pilot experiments examining their dose-dependant effects on neuronal plasma membrane integrity and [ATP] (pilot data not shown). STS and Oligomycin A were dissolved in DMSO to a final [DMSO] <0.01%, and solutions were made fresh daily. Chemicals were purchased from Sigma unless otherwise indicated (Sigma-Aldrich, St. Louis, MO).

### Annexin V Assay and Confocal Microscopy

Annexin V expression was measured following 24 hrs of treatment using Annexin V-FITC Apoptosis Detection Kits (Enzo Life Sciences, Plymouth Meeting, PA) as per the manufactures instructions. Fixed samples were imaged on an Olympus FV1000 scanning confocal microscope, using 488 nm (FITC) and 405 nm (DAPI) laser lines (Olympus, San Diego, CA). For data collection, the parameters of the microscope such as light intensity, exposure time, camera gain, etc., were determined for the brightest fluorescing sample and standardized for subsequent samples. Experiments were repeated 4 times and for co-localization analysis, five random sections from each study group were taken at 20× magnification using AxioVision (Carl Zeiss, Thornwood, NY), and the percentage of neurons staining positive for Annexin V was determined by the ratio of FITC-stained cells to DAPI-stained nuclei. Metamorph (Molecular Devices, Sunnyvale, CA) image analysis software was used to count fluorophore-positive stained cells/DAPI-positive cells. In other experiments total Annexin V fluorescence was assessed in 96-well microplates (Corning) seeded at a density of ∼50,000 cells per well. Samples were treated for 24 hrs and then analyzed on a Bio-Tek PowerWave 340 microplate spectrophotometer (Bio-Tek, Winooski, VT, Ex/Em: 485/530 nm) using Gen 5 software (Bio-Tek) within one hour of staining.

### ATP Luciferase Assay

Total ATP content [ATP] was assessed in solid-bottom, black 96- or 384-well microplates (Corning) using PerkinElmer ATPlite Luminescence Assay System kits as specified by the manufacturers protocol (PerkinElmer, MA, USA) and a Bio-Tek PowerWave 340 microplate spectrophotometer (Bio-Tek, Winooski, VT). Equal numbers of cells were seeded into each well (∼50,000 and 5,000 cells/well for 96- and 384-well plates, respectively) and [ATP] was assessed following 0, 2, 6, 12, 18, 24, 36, and 48 hrs treatment. Standard curves were generated using serial dilutions of a known ATP standard provided in each kit. The sensitivity of the detector was calibrated to the luminescence of the highest [ATP] standard in each experiment. Results were normalized to ATP luminescence recorded from control cells assayed at *t* = 0 hours. Microplate ATP luciferase experiments were repeated 5 times in parallel, and each plate contained at least 16 replicate wells of each treatment group. Blank wells and cell-free wells containing each treatment perfusate were also included on each plate, and the final data is corrected for these factors.

### Gel Electrophoresis DNA Fragmentation Assay

Samples grown in 150 cm^2^ culture flasks were treated as indicated in the experimental design section (above) for 0, 12, 24 or 48 hrs. Following treatment, cells were rinsed twice in PBS and then rapidly homogenized in ice-cold PBS by pipetting and vortexing for up to 60 seconds. Purified DNA was extracted from the resulting homogenates using a Qiagen DNAeasy Blood and Tissue kit as specified by the manufacturers protocol (Qiagen, Valencia, CA), and quantified by nanodrop (Thermo Sci., Wilmington, DE) Equal quantities of DNA were heated at 65°C for 10 mins, and then loaded onto a standard 1.5% agarose gel containing 0.5 mg/ml ethidium bromide. Electrophoresis was performed at 120V for ∼20–40 mins at room temperature to separate DNA fragments by weight. Specific bands were visualized via UV trans-illumination using a BioRad Chemilab XRS+ gel-dock system (BioRad, Hercules, CA). Densitometry was performed and images were analyzed using BioRad imaging software (BioRad). Experiments were repeated 3 times.

### Protein Extraction and Western Blots

Samples grown in 150 cm^2^ culture flasks were treated for 6 hrs because this was the latest time point at which high quality proteins could be extracted from all treatment groups (pilot data not shown). Following treatment, samples were rinsed twice with PBS and detached from the matrix with a cell scrapper into ice-cold PBS. The resulting cell suspensions were centrifuged at 250×*g* for 5 mins at 4°C, the supernatant was aspirated away, and cells were re-suspended in cell lysis buffer. Samples were then homogenized by vortexing for 60 seconds and proteins were extracted by incubation in lysis buffer with mixing at 4°C for 45 mins, followed by centrifugation for 10 mins at 14,000×*g* at 4°C. Supernatants were taken as whole cell lysates and protein concentration was measured using a bicinchoninic acid kit, according to the manufacturer’s instructions (Sigma).

For Western blot analysis, equal amounts of protein (40 µg/well) were separated on 4–12% precast NuPAGE bis-Tris SDS-PAGE gels (Invitrogen) and transferred to polyvinylidene difluoride membranes (Immobilin-P; Millipore, Bedford, MA). Western blots were performed with antibodies against α-actin, AIF, beclin 1, cleaved caspase 3, lamin A, light chain 3 (LC3), mTOR, and cleaved Poly (ADP-ribose) polymerase (PARP) (1∶1,000, Cell Signaling, Danvers, MA); Bcl-2, cytochrome C, or JNK3 (1∶200, Santa Cruz Biotechnology, Santa Cruz, CA). Specific bands were visualized after incubation with the respective secondary antibodies (1∶2000) using enhanced chemiluminescense (GE Healthcare/Amersham Biosciences, Buckinghamshire, UK). Following chemiluminescent detection, membranes were stripped of antibodies by incubation in 50 ml of TRIS stripping buffer with 2% SDS and 0.7% β-mercaptoethanol (pH 6.8) for 30 minutes at 50°C, and then subsequently re-probed for other proteins. This process was repeated such that each protein was assayed once per membrane, allowing for direct comparison of protein changes on the same blot (Note: cleaved PARP and LC3 assays were performed on a separate group of blots and therefore have different associated actin controls). Densitometry of Western blots from each experimental group were obtained (*n* = 3 gels for each), and absolute values were normalized to α-actin expression on the same blot. Results were analyzed in arbitrary units, comparing each value with that obtained from each respective α-actin measurement on each blot, and results are expressed as fold-change relative to untreated controls run simultaneously.

### RNA Extraction and Quantification and RT-PCR

The expression of selected genes was determined by RT-PCR analysis. Following treatment, cells grown in tissue culture flasks were rinsed and detached in ice-cold PBS. Cell suspensions were centrifuged at 250×*g* for 5 mins at 4°C with an Eppendorf 5810 R centrifuge (Eppendorf, San Diego, CA), the supernatant was discarded and cells were re-suspended in cell lysis buffer with 1% β-mercaptoethanol and lysed by vortexing. Total RNA was extracted using a Clontech Nucleospin RNA II kit with on-column DNAase step (Macherey-Nagel, Bethlehem, PA). RNA quantity and integrity (RIN) was assessed with an Agilent 2100 Bioanalyzer and RNA chips (Agilent, Santa Clara, CA). cDNA was synthesized from total RNA using the Superscript III First Strand kit (Invitrogen), according to manufacturer’s instructions. Briefly, 1 µg of RNA was annealed to 1 µl of OligodT at 65°C for 5 mins. cDNA was synthesized by cycling at 50°C for 50 minutes, and 85°C for 5 minutes. The reactions were treated with 1 ul RNase H at 37°C for 20 minutes, and cDNA quantity and quality was verified using a ND-1000 spectrophotometer (Thermo Sci.).

For PCR amplification of cDNA, gene expression was quantified using a Power SYBR green PCR kit (Life Tech., Carlsbad, CA) according to manufacturer’s instructions. Briefly, 20 µl reaction mixtures were prepared containing specific primers (1 µl each of fwd and rev primers), 1 µl of cDNA (1.5 µM) and the SYBR green master mix. Experiments were performed under the following conditions: 95°C for 10 min followed by 40 cycles of 95°C/15 sec, 60°C/60 sec, then 95°C/15 sec, 60°C/15 sec, and 95°C/15 sec, on an AB 7600 RT-PCR machine (Life Tech.). Changes in gene expression were measured by comparing CT values to the housekeeping gene, β-actin. Primers were obtained from ValueGene (San Diego, CA) and the oligonucleotide primer sequences used were as follows. atg7 (fwd: 5′-GTGCCTCACCAGATCCGGGGTT-3′, rev: 5′-AGGAAGGTGAATCCTTCTCGCTCG-3′), β-actin (fwd: 5′-GTGACGTTGACATCC-GTAAAGA-3′, rev: 5′-GCCGGACTCATCGTACTCC-3′) mtor (fwd: 5′-AAGCCCCAGCT-CGGAGGTGT-3′, rev: 5′-AACGGCCAGGGAGCGGGTAT-3′), and PI3K (fwd: 5′-AACCCCACCGTGAGGCGCTA-3′, rev: 5′-AGCAAATCCTCATCATCGGCCTGC-3′).

### Transmission Electron Microscopy

Samples in 35 mm #0 thickness culture dishes were fixed with a 37°C solution of 2% paraformaldehyde, 2.5% glutaraldehyde (Ted Pella, Redding, CA) in 0.1 M sodium cacodylate (pH 7.4), and transferred to room temperature for 10 mins, and then incubated for an additional 30 mins on ice. Fixed cultures were rinsed 3 times for 3 mins each with 0.1 M sodium cacodylate plus 3 mM CaCl_2_ (pH 7.4) on ice and then post-fixed with 1% osmium tetroxide (Ted Pella), 0.8% potassium ferrocyanide, and 3 mM CaCl_2_ in 0.1 M sodium cacodylate (pH 7.4) for 60 mins, and were then washed 3 times for 3 mins with ice-cold distilled water. Cultures were finally stained overnight with 2% uranyl acetate at 4°C, dehydrated in graded ethanol baths, and embedded in Durcupan resin (Fluka, St. Louis, MO). Ultrathin (70 nm) sections were post-stained with uranyl acetate and lead salts, and evaluated by a JEOL 1200FX transmission electron microscopy operated at 80 kV. Images were recorded on film at 6,000× magnification. The negatives were digitized at 1,800 dpi using a Nikon Cool scan system, giving an image size of 4033×6010 pixel array and a pixel resolution of 2.35 nm [29]. Images of 20 cells or organelles were taken from each experimental condition. All TEM experiments were repeated at least twice. TEM analysis of cytoplasmic vacuole density, and mitochondrial and nuclear morphology was performed using standard stereology techniques by measuring at >10 randomly chosen locations from each experimental replicate.

### Statistics

Data were analyzed using a two-tailed Student *t*-test or one-way analysis of variance (ANOVA), followed by Dunnet’s post-test. For all statistical analysis, significance was indicated if *P*<0.05 assuming two groups had an equal variance. Statistical analysis was performed using Prism software (GraphPad, San Diego, CA).

## Results

### IS Induces Extensive Vacuolization of the Cytoplasm, [ATP] Depletion, and Annexin V Staining

**Figure 1:**
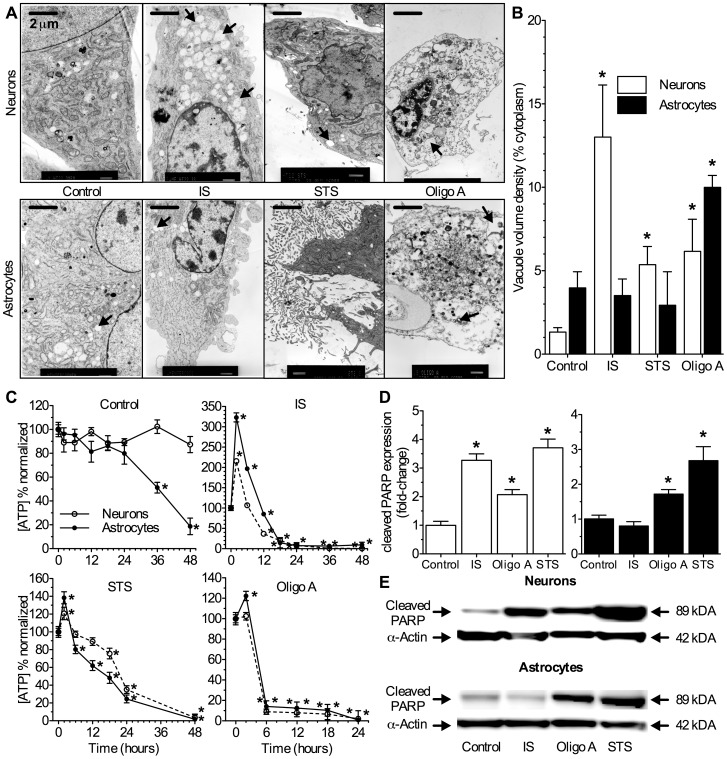
IS induces extensive autophagic vacuolization of neuronal cytoplasm and depletes [ATP] in neurons and astrocytes. IS-treated neurons and oligomycin-treated neurons and astrocytes exhibit extensive cytoplasmic vacuolization, organelle digestion, and [ATP] changes characteristic of autophagy. (**A**) Sample TEM images of neurons (upper panels) and astrocytes (lower panels) treated as indicated for 24 hrs. Arrows indicate vacuoles. (**B**) Summary of vacuole density in the cytoplasm by volume. TEM experiments were repeated 2–3 times and 10–20 cells were examined from each treatment group. (**C**) Summary of change in [ATP] with time from neurons (open symbols) and astrocytes (closed symbols) treated as indicated through 48 hrs from >10 different experiments. (**D**) Summary of fold-change in protein expression from western blot analysis of PARP cleavage in samples treated for 6 hrs. Changes were normalized to α–actin expression in the same sample. (**E**) Sample western blots from (D). Blots are representative of 3 separate experiments. Data are mean ± SEM. Asterisks (*) indicate significant difference from untreated controls (*p*<0.05). Treatments: control (DMEM/F12), ischemic solution (IS), 2.5 µm staurosporine (STS), and 10 µm oligomycin A (Oligo A).

We first examined whole cell morphology following 24 hrs of treatment using TEM. Control cells had intact nuclei and plasma membranes, with healthy organelles throughout the cytoplasm (Fig. 1A, images are representative of 20 cells examined per treatment in 3 experiments). Neurons maintained >85% [ATP] through 48 hrs, whereas astrocyte [ATP] was >80% of baseline controls (i.e. time = 0 hrs) through 24 hrs of treatment, and then declined to 51.2±4.5% and 18.7±6.9% at 36 and 48 hrs, respectively (Fig. 1A–C, *n* = 20–30 for each treatment and time point examined). IS-treated neurons, but not astrocytes, had increased autophagic vacuolization of the cytoplasm (Fig. 1A arrows); and both cell types exhibited catabolic digestion of organelles and plasma membrane degradation (Fig. 1A&B). A transient increase in [ATP] was observed during the first 2–12 hrs following IS-treatment onset. This increase was greater in astrocytes than in neurons and [ATP] increased ∼3- and 2-fold in these two populations, respectively. [ATP] declined rapidly to <10% of baseline by 18 hrs of IS treatment in both cell types (Fig. 1C). [ATP] depletion has been linked to the activity of Poly (ADP-ribose) polymerase (PARP), a nuclear protein that utilizes cellular ATP to repair single-strand DNA breaks [30], [31]. In systems where DNA damage is extensive and apoptosis is initiated, PARP is cleaved by caspase-3 and thereby inactivated. As such, PARP cleavage is an indicator of cell death pathways in tandem with [ATP] measurements. In our experiments the expression of cleaved PARP protein increased ∼3-fold in IS treated neurons following 6 hrs of treatment, while cleaved PARP expression did not change in IS treated astrocytes at this time point (Fig. 1D&E, *n* = 3).

In apoptosis-positive controls, STS-treated cells were extensively fragmented and phenotypically in the late stages of apoptosis (cleavage-mediated deconstruction of organelles, plasma membranes, and whole cells was apparent) (Fig. 1A) [8]. Similar to IS treated samples, [ATP] was transiently elevated in the first 4 hrs after treatment-onset and was maintained at >60% of baseline through 12 hrs of STS treatment in astrocytes and 18 hrs in neurons (Fig. 1C). This is consistent with the induction of apoptosis, the execution of which requires ATP [26]. In support of this, the expression of cleaved PARP increased ∼3 to 4-fold in cells treated with STS (Fig. 1D&E). Conversely, autophagy-positive cells treated with oligomycin A had a phenotype of late-stage autophagy [8], including extensive cytoplasmic vacuolization and digestion of organelles (Fig. 1A&B). The induction of an autophagy-like phenotype in these cells correlated temporally with a rapid depletion of [ATP] within 6 hrs of treatment, and a modest (∼20%) and transient increase in [ATP] was observed in astrocytes but not neurons following 2 hrs of treatment onset and prior to this rapid energy depletion (Fig. 1C). The expression of cleaved PARP protein was also reduced in these samples relative to IS or STS treated neurons or STS treated astrocytes (Fig. 1D&E).

**Figure 2:**
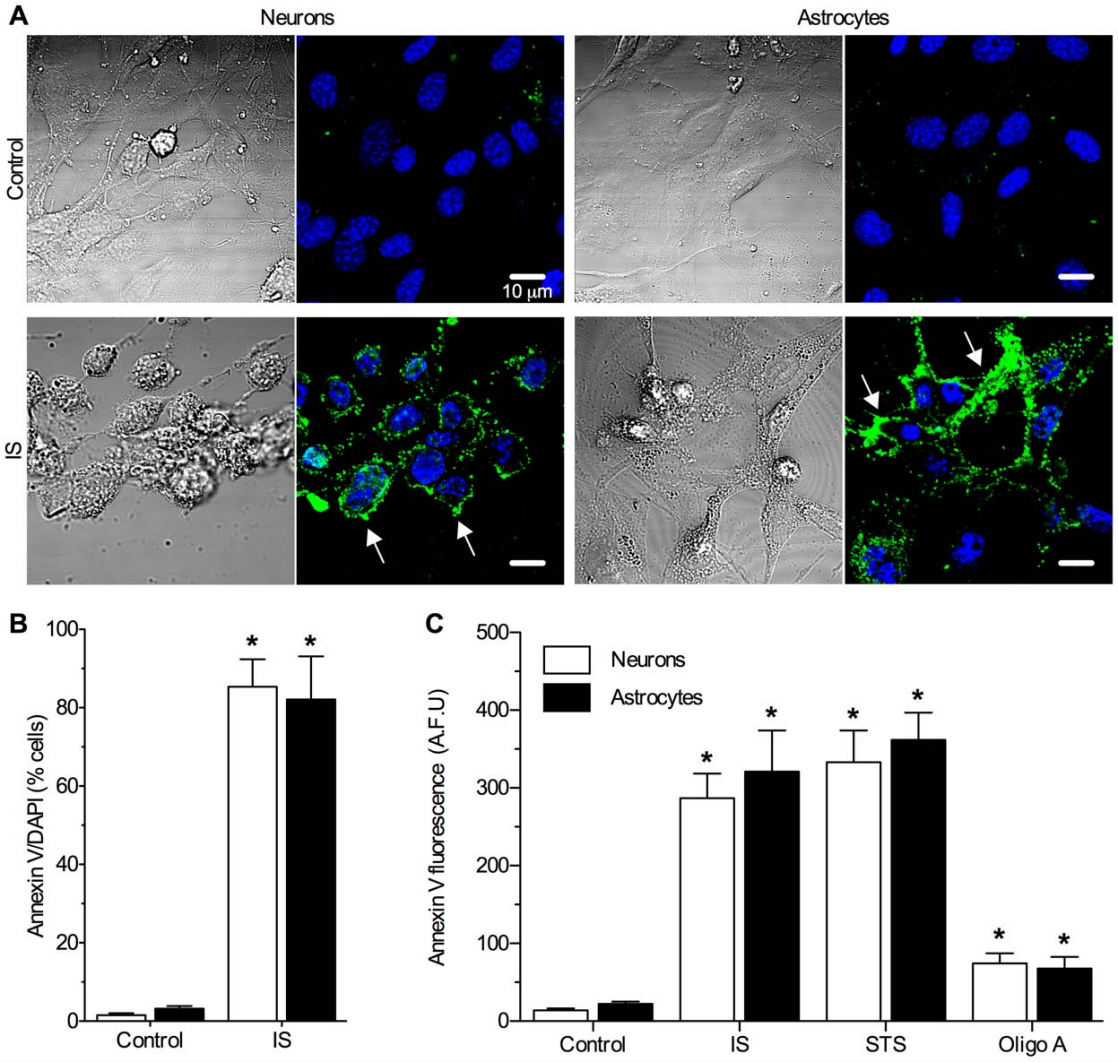
IS induces apoptotic Annexin V translocation in neurons and astrocytes. (**A**) Sample paired DIC (left panels) and Annexin V and DAPI (green and blue fluorescence, respectively; right panels) confocal microscopy images of neurons and astrocytes treated as indicated for 24 hrs. Images are representative of 4 separate experiments. (**B**) Summary of the ratio of Annexin V-positive stained cells to DAPI-stained nuclei. (**C**) Summary of Annexin V fluorescence from neurons or astrocytes treated in 96-well microplates as indicated for 24 hrs. Data are mean ± SEM. Asterisks (*) indicated significant difference from untreated controls (*p*<0.05).

To directly examine the induction of apoptosis, we also assayed the translocation of phosphatidylserine residues that bind Annexin V to the extracellular plasma membrane surface [32], [33]. Following 24 hrs treatment, significant Annexin V staining was not observed in control cells and only 1.5±0.5% of neurons and 3.2±0.7% of astrocytes stained positive for Annexin V (*n* = 4 each, Fig. 2A&B). In contrast, following 24 hrs IS treatment Annexin V staining was apparent on the extracellular membrane surface of the vast majority of neurons and astrocytes (85.4±7.2% and 82.1±11.0%, respectively). In other experiments we examined total Annexin V fluorescence from neurons or astrocytes seeded in 96-well microplates following 24 hrs of treatment as indicated. In good agreement with our confocal microscopy analysis, minimal Annexin V fluorescence was detected in control cells (Fig. 2C, *n* = 20 replicates per treatment group from 4 experiments each). IS treatment increased Annexin V fluorescence in neurons and astrocytes by ∼22- and 14-fold, respectively. Similarly, STS treatment increased Annexin V fluorescence ∼26- and 16-fold in neurons and astrocytes, respectively; whereas Oligomycin A treatment induced comparatively mild increases in Annexin V fluorescence in both cell types.

### IS Induces Mitochondrial Fission and Reduced Cristae Density

**Figure 3:**
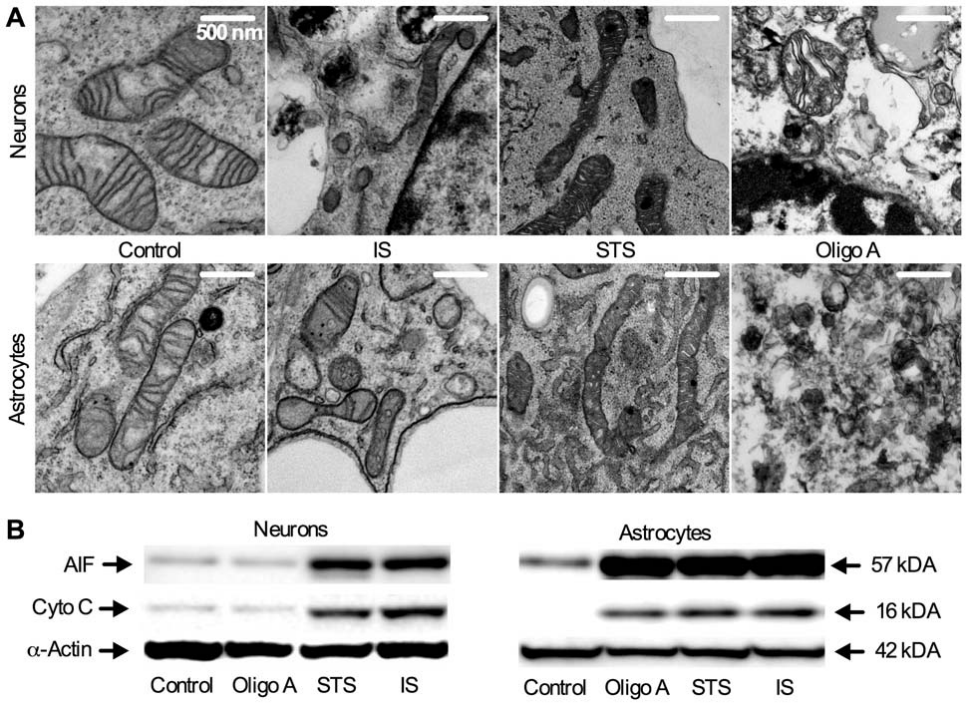
IS causes mitochondrial fission and membrane rupture. (**A**) Sample TEM images of mitochondria from neurons (upper) and astrocytes (lower) treated as indicated for 24 hrs. (**B**) Sample western blots of apoptosis inducing factor (AIF) and cytochrome C (Cyto C) release from samples treated as indicated for 6 hrs. Blots are representative of 3 separate experiments.

We next examined mitochondrial morphology, and also cytochrome C and apoptosis inducing factor (AIF) release as common indicators of apoptosis-mediated mitochondrial cleavage/rupture. Control mitochondria were smooth with extensive and even cristae networks, and AIF and cytochrome C expression were minimal (Figs. 3A&B, S1A–D). IS-treated mitochondria were irregular in shape with reduced cristae (Figs. 3A, S1D). These mitochondria underwent fission and were ∼25% smaller than controls, while the total number of mitochondria/cell increased ∼55–60% in both cell types (Fig. S1C). Mitochondrial volume density was reduced ∼50% in IS-treated neurons but was unchanged in astrocytes (Fig. S1C), likely because astrocyte mitochondria were more swollen and balloon-like than those in neurons (Fig. 3A). In both cell types cristae density and perimeter were decreased by ∼50–70% (Fig. S1D). AIF expression increased ∼6-fold in both cell types, while cytochrome C expression increased >3- and 6-fold in neurons and astrocytes, respectively (*n* = 3, Figs. 3B, S1A&B), indicating that mitochondrial membrane rupture had occurred.

Mitochondria from STS treated cells underwent fusion and were 25–40% longer than controls and irregular in shape (Figs. 3A, S1C) [34], while AIF and cytochrome C expression increased to the same degree as in IS (Figs. 3A–B, S1A, B&D). The total number of mitochondria per cell decreased ∼70% in astrocytes and tended to be reduced in neurons, although this trend did not reach significance (Fig. S1C). Stereological analysis of STS treated samples revealed decreased cristae density relative to controls in astrocytes but not neurons; however, cristae perimeter increased ∼3-fold in both cell types. Cristae density is normalized to total outer mitochondrial membrane (OMM) surface area, whereas cristae perimeter provides an absolute number of total cristae. Therefore, the large increase in cristae perimeter despite decreased or maintained cristae density in STS treated neurons and astrocytes indicates that mitochondria are markedly swollen, supporting our conclusion that mitochondrial fusion has occurred. Oligomycin A treated mitochondria were >50% smaller than controls and cristae density was reduced 10-fold in neurons and >75% in astrocytes (Figs. S1C&D). Mitochondria in these samples were highly digested and the total number of mitochondria per cell was >90% lower in both neurons and astrocytes relative to controls (Fig. S1C). AIF and cytochrome C expression were not markedly changed in neurons, but the expression of these proteins increased ∼4 fold each in astrocytes, possibly due to more advanced catabolism in astrocytes at this time-point facilitating the release of these proteins (Figs. 3B, S1A&B).

### IS Treated Nuclei Exhibit Chromatin Condensation and Nuclear Membrane and DNA Cleavage

**Figure 4:**
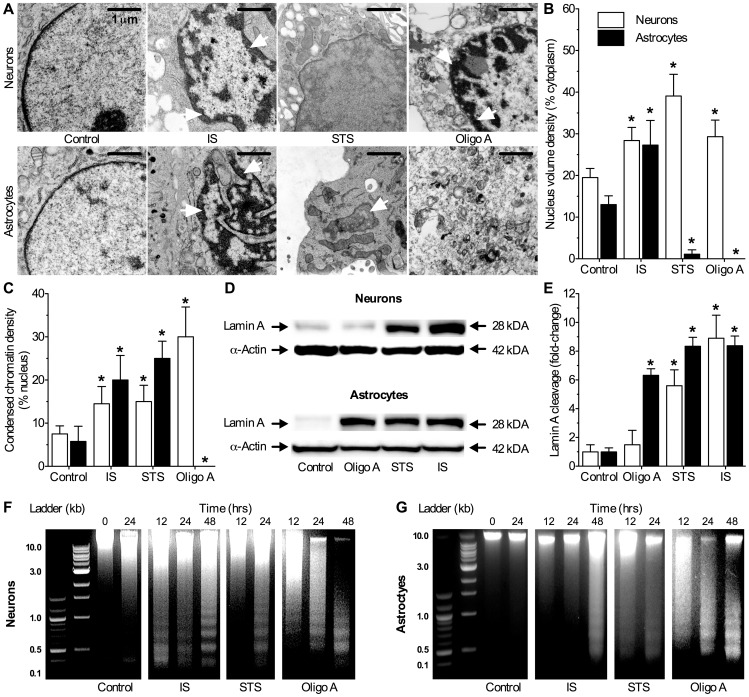
IS-treated nuclei exhibit apoptotic chromatin condensation, nuclear envelope fragmentation into apoptotic bodies, and laddered DNA cleavage. (**A**) Sample TEM images of nuclei from neurons (upper panels) and astrocytes (lower panels) treated as indicated for 24 hrs. White arrows indicate condensed chromatin beads and fragmenting nuclei. (**B**) Summary of nuclear volume density from samples in (A). (**C**) Summary of chromatin volume density from samples in (A). Note: nuclei were too fragmented in oligomycin A-treated astrocytes for quantification. (**D**) Sample western blots of lamin A cleavage in samples treated as indicated for 6 hrs. Blots are representative of 3 separate experiments. (**E**) Summary of fold-change in protein expression from (D) normalized to α–actin expression in the same sample. (**F&G**) Sample conventional agarose gel electrophoresis gel images of DNA fragmentation from neurons (F) and astrocytes (G) treated as indicated for 0, 12, 24, or 48 hrs. Gels are representative of 4 separate experiments. Data are mean ± SEM. Asterisks (*) indicate significant difference from untreated controls (*p*<0.05).

AIF acts on the nucleus to induce chromatin aggregation, DNA cleavage, and beading and cleavage of the nuclear membrane into apoptotic bodies [8]. Since AIF was released from ruptured mitochondria in IS-treated cells, we next examined nuclear phenotypes. Control nuclei appeared smooth and even with an oval shape and took up 19.5±2.2 and 13.0±2.1% of the total cytoplasmic space in neurons and astrocytes, respectively (Fig. 4A&B). Chromatin was largely aggregated in nucleosomes and chromatin density along the nuclear membrane was <8% in both control groups (Fig. 4A&C). In contrast, IS-treated cells exhibited a phenotype of mid- to late-stage nuclear apoptosis [8]: nuclei were irregular in shape and took up 50–100% more of the total cytoplasmic space than in controls (Fig. 4A&B). Furthermore, IS-treated nuclei exhibited extensive bead-like chromatin condensation along the nuclear membrane and chromatin density increased 2-fold in neurons and 4-fold in astrocytes (Fig. 4A&C).

In addition to morphology we also examined cleavage of lamin A, a component of the nuclear membrane that must be cleaved in order for late-stage nuclear apoptosis (i.e. cleavage and fragmentation of the nuclear membrane into distinct apoptotic bodies) to proceed [8]. In IS-treated cells lamin A expression increased ∼8-fold relative to controls (Fig. 4 D&E). Similarly in apoptotic cells treated with STS, lamin A increased 6 to 8-fold and nuclei were fragmented and in many samples had been entirely dismantled (Fig. 4A, D&E). In cells with identifiable nuclear structures, nuclear volume density was decreased and condensed chromatin density increased 2-fold in neurons and 5-fold in astrocytes (Fig. 4A–C). Finally, in pro-autophagy oligomycin A treated neurons nuclei were extensively dismantled, while in astrocytes nuclear degradation was near total and nuclei and chromatin could not be identified in a sufficient number of samples for statistical analysis. In oligomycin A treated neurons in which nuclear structures could be identified, condensed chromatin density increased 4-fold relative to controls. Lamin A cleavage increased ∼6-fold in astrocytes treated with oligomycin A, but was unchanged in neurons (Fig. 4D&E).

AIF and other pro-apoptotic factors released from mitochondria induce oligonucleosomal cleavage of DNA into discrete low molecular weight DNA fragments [8]. We used conventional agarose gel electrophoresis to determine the extent and pattern of DNA fragmentation and results from this analysis correlated with our nuclear morphology examinations. DNA isolated from untreated controls was uncleaved, whereas IS treated neuronal DNA exhibited apoptotic cleavage at 12, 24 and 48 hrs (*n* = 3 for each cell type, Fig. 4F&G). DNA from STS or oligomycin A treated neurons exhibited similar apoptotic cleavage at these time points. In astrocytes, IS treatment caused DNA cleavage only at the later time point (48 hrs), whereas STS and oligomycin A induced more extensive fragmentation within 12–24 hrs.

### IS Upregulates Autophagy and Apoptotic Pathways

To better understand the pathways initiated by IS we next examined changes in the expression of mRNA and proteins related to cellular autophagy and apoptosis. Apoptosis, mediated either by cytochrome C release from ruptured mitochondria or immune receptor-mediated JNK3 activation, induces caspase-dependant cascades; which are molecularly characterized by caspase 3 activation and inhibited by increased Bcl-2 expression [35], [36]. Autophagy is induced by metabolic starvation and is molecularly characterized by increased beclin 1 expression, an increase in the ratio of microtubule-associated protein 1 light chain 3-I to light chain 3-II isoform expression (LC3I/LC3II) [37], and also the activation of PI3K and atg7. Autophagy is inhibited by the mTOR pathway, which prevents vacuole formation [38].

**Figure 5:**
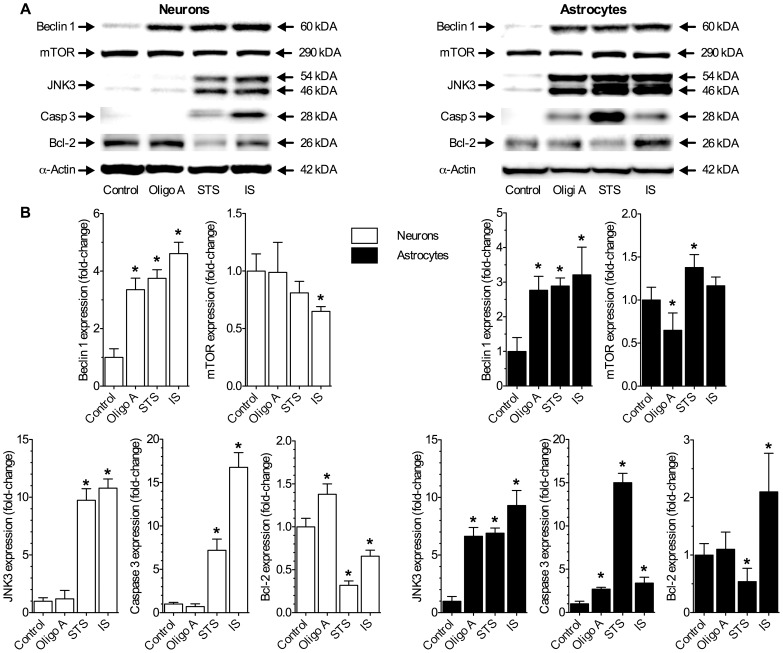
IS upregulates autophagy and apoptotic pathways in neurons and astrocytes. (**A**) Sample Western blots of autophagy- and apoptosis related protein expression from neurons (left panels) and astrocytes (right panels) treated as indicated for 6 hrs. (**B**) Summary of fold-change in protein expressions from (A) normalized to α–actin expression in the same sample. Data are mean ± SEM from 3 separate experiments for each protein, cell type, and treatment. Asterisks (*) indicate significant difference from untreated controls (*p*<0.05).

In control samples only Bcl-2 and mTOR were expressed (*n* = 3 for each treatment, Fig. 5A&B). In contrast, STS-treated cells had a molecular profile typical of apoptosis, including 7 and 15-fold increases of caspase 3 and JNK3 expression, respectively; and 60% lower Bcl-2 expression (Fig. 5A&B). Autophagy was largely inhibited in these cells and although beclin 1 expression increased ∼3–4 fold relative to controls, PI3K and atg7 mRNA expression were unchanged or slightly decreased and mTOR expression was unchanged or mildly elevated (Figs. 5A&B, S2A–C), and LC3II/LC3I ratio increased mildly in neurons and astrocytes (Figs. 5C&D). Conversely, oligomycin A treated cells had a largely autophagic phenotype, including increased PI3K and atg7 mRNA expression in both cell types and decreased mTOR mRNA expression in neurons and decreased mTOR protein expression in astrocytes, suggesting more rapid upregulation of autophagy in astrocytes relative to neurons. Beclin 1 expression increased ∼3-fold in both cell types and the LCII/LCI ratio 2.5-fold in astrocytes and 4-fold in neurons; whereas Bcl-2 expression increased ∼50% in neurons and caspase 3 and JNK3 were not extensively activated. The inhibition of apoptosis was weaker in astrocytes, such that Bcl-2 was not increased and cytochrome C and JNK3 expression were higher relative to controls.

IS treated cells had a molecular profile that was a mixture of autophagy and apoptotic phenotypes. In addition to cytochrome C, AIF, and caspase-dependant lamin A cleavage (Figs. 3B, 4D–E, &S1A–B), the induction of apoptosis by IS was supported by 10-fold increases in JNK3 expression and 17- and 3-fold increases in caspase 3 expression in neurons and astrocytes, respectively (Fig. 5A&B). Furthermore, Bcl-2 expression was 40% lower in neurons, but increased 2-fold in astrocytes. Our results also indicate the induction of autophagy by IS treatment. In both cell types IS increased beclin 1 expression 3 to 4-fold and PI3K and atg7 mRNA expression increased 2 to 4-fold. LC3II/LC3I ratios were increased ∼50% in both cell types, and mTOR mRNA and protein expression were slightly decreased or unchanged by IS treatment.

## Discussion

We demonstrate that our ischemic penumbral mimic solution (1) induces a cell death phenotype of mixed apoptosis and autophagy in cultured murine neuronal and astrocytic cell lines, and (2) is more deleterious to astrocytes than neurons. This phenotype is apparent at the whole-cell, organelle, bioenergetic, and molecular levels, and reasonably mimics the ischemic penumbra *in vivo*; where extensive activation of apoptosis and autophagy have been reported [3], [4], [5], [39], [40]. The induction of apoptosis in IS-treated cells is confirmed at the morphological level by the occurrence of cellular shrinkage and reduced mitochondrial cristae density. The nuclear phenotype in IS-treated cells is also typical of apoptosis, including chromatin condensation and cleavage of the nuclear membrane into distinct apoptotic bodies; accompanied by laddered DNA cleavage [8], [41]. Bioenergetically, we observe a large and transient increase in [ATP] during the first 2–6 hrs of IS-treatment, an effect that has also been described in the penumbra *in vivo* [42]. Importantly, [ATP] is then maintained at >50% of baseline through the first 12–18 hrs of IS-treatment, enabling the ATP-dependant progression of apoptosis [8], [26], [43], and consistent with STS treated cells. Finally, we observe multiple molecular indicators of apoptosis, including Annexin V binding and increased expression of apoptosis-related AIF, cytochrome C, lamin A, JNK3, cleaved PARP, and caspase 3 [44], [45], [46]. These changes are similar to those observed in apoptosis-positive controls, although morphologically, STS-treated cells are in a more advanced stage of apoptosis than IS-treated cells and exhibit total dismantling of some cells and organelles. In addition to our present findings, previous studies have demonstrated that IS-treatment induces plasma membrane degradation via matrix-metalloproteinase activation and increases reactive oxygen species generation and immune- and apoptosis-related mRNA expression in cultured murine cells [18], [20], [21]. Taken together these data strongly support the induction of apoptosis in cultured neurons and astrocytes by IS-treatment.

In addition to apoptosis we report evidence of extensive autophagy in both cell types treated with IS. Autophagy is a form of self-catabolism wherein cells digest their own organelles and other cytoplasmic contents to recover energy and substrates and preserve [ATP] [47]. Morphologically, autophagy is indicated in IS-treated neurons by extensive cytoplasmic vacuolization. Interestingly, the extent of cytoplasmic vacuolization in IS-treated neurons is greater than in apoptosis- or autophagy-positive controls treated with STS or Oligomycin A. The reason for this difference is unclear; but may be due to a variety of factors. The activation of apoptosis and autophagy often overlap in the same cell [6], and the degree to which either pathway predominates is determined by the stressors applied [47]. STS-treatment activates caspase-3 mediated apoptosis [23], whereas IS likely activates numerous other cell death pathways that contribute to the induction of autophagy and lead to more extensive cytoplasmic vacuolization. Conversely, Oligomycin A treatment causes rapid depletion of >90% [ATP] within 6 hours of treatment, whereas in IS-treated cells [ATP] is not depleted to this degree until >18 hours. This delayed decay of [ATP] may prolong the period during which autophagic catabolism is employed to recover energetic substrate, thus increasing the extent of cytoplasmic vacuolization in these samples.

Along with extensive cytoplasmic vacuolization we also observe upregulation of the key autophagy-related protein beclin-1 in both cell types, along with increases in the ratio of LC3II/LC3I protein isoforms and also increased expression of atg7 and PI3K mRNA. In addition, anti-autophagy mTOR mRNA (astrocytes) and protein expression (neurons) decrease during IS treatment. These observations are consistent with autophagy-positive (oligomycin A treated) controls in which similar changes in beclin-1, atg7, PI3K, LC3II/LC3I, and mTOR expression occur. Taken altogether, our results support a phenotype of mixed apoptosis and autophagy in cultured cell lines treated with IS. Our present study is the first comprehensive examination of cell state in an *in vitro* ischemic penumbral mimic suitable for HTS, and our results also agree with bioenergetic and molecular examinations in the penumbra *in vivo*, which indicate the occurrence of both autophagy and apoptosis [6], [10], [14], [34], [39], [42], [46], [48], [49], [50], [51], [52], [53].

In addition to apoptosis and autophagy, necrosis is recognized as the 3^rd^ major cell death modality in eukaryotic cells [8], [54]. Necrosis is typically induced by deleterious alterations in the extracellular environment and hallmarks of necrosis include cell swelling, loss of membrane integrity and uncontrolled cell lysis. Necrotic cell death is characteristic of neurons located in the ischemic infarct core in stroke pathology, where [ATP] declines within minutes of insult onset, preventing the execution of ATP-dependant programmed apoptosis (e.g. regulated organelle, DNA, and plasma membrane cleavage) [26], [55]. Concomitantly, uncontrolled osmolyte influx leads to rapid cell swelling and membrane rupture, which permits the leakage of inflammatory components from ruptured cells [8], [56], [57]. These events contribute to the initial formation and spread of the ischemic penumbra [3]; however, penumbral cell death is currently thought to proceed via programmed cell death pathways, with minimal contribution from necrosis [3], [6], [14], [20], [50], [53]. In good agreement, our present results do not support the occurrence of necrosis in neurons treated with our ischemic penumbral model. IS-treated neurons and astrocytes do not exhibit rapid depletion of [ATP], DNA and organelle cleavage occurs in a programmed, apoptotic fashion, and cell swelling is not observed. Furthermore, in a recent study examining the effects of IS-treatment on neuronal membrane integrity we reported that plasma membranes were dismantled in a regulated fashion via the activity of matrix metalloproteinases, consistent with the execution of apoptosis [21].

A key observation in our study is that astrocytes are more sensitive to metabolic and IS-mediated stress than neurons and exhibit a stronger autophagic response to these stresses. In support of this: 1) vacuoles are apparent in the cytoplasm of astrocytes in all treatment groups; 2) in IS-treated astrocytes [ATP] is 2-fold higher than in neurons at each time point through the first 12 hrs of treatment; 3) PARP is not cleaved following 6 hrs treatment in astrocytes but the expression of cleaved PARP proteins increases 3-fold in neurons; 4) caspase-3 activation is 5-fold lower in IS-treated astrocytes than in similarly treated neurons; and 5) autophagic Bcl-2 protein expression is increased 2-fold in IS-treated astrocytes. In addition, metabolic stress induced by oligomycin A induces apoptosis in addition to autophagy in astrocytes: AIF, cytochrome C, lamin A, JNK3, cleaved PARP, and caspase 3 expression are all elevated here but not in similarly-treated neurons; indicating metabolic inhibition represents a greater stress to astrocytes and activates apoptotic pathways in addition to autophagy. Furthermore, organelles in oligomycin A treated astrocytes are markedly degraded at 24 hrs relative to neurons and total mitochondrial number decreases >90% relative to controls, while nuclei can only be indentified in one of the samples. Together, these results indicate that astrocytes are more sensitive to ischemic and metabolic stresses than neurons. Other groups have also reported that astrocytes are more susceptible to metabolic or ischemic injury than neurons; and this heightened sensitivity to metabolic stress may contribute significantly to penumbral expansion since astroglial dysfunction plays a key role in the progression of ischemic injuries *in vivo* and because there is evidence that astrocytic demise precedes and directly contributes to neuronal demise in ischemia [58], [59].

It is important to note that the ischemic penumbra is a complex system of interacting pathways and cell types and that *in vitro* examinations in immortalized cell lines are an incomplete mimic of the brain *in situ*, or the penumbral milieu. In particular, sub-populations of neurons and astrocytes interact with each other and with vascular and endothelial cells *in vivo* (the “neurovascular unit”), and numerous inflammatory and blood perfusion differences exist within the largely heterogeneous penumbra that significantly impact cellular viability within and between these sub-populations [60], [61]. The spread of the penumbra is variable depending on numerous, poorly understood factors and interactions between microvascular beds and nearby neurons and astrocytes may lead to the formation of “micro-cores” and “micro-penumbras” within the greater penumbral region itself. Within these regions cellular responses vary greatly depending on the degree of perfusion and inflammation, including variable regions of peri-infarct spreading depression, stress gene and protein synthesis responses, and potential for cellular recovery from insult [60], [61]. Nonetheless, strategies to screen high volumes of drugs, molecules, and compounds for cytoprotective interactions against ischemic insult offer the potential for rapid discovery of pharmacoprotective agents, and such screens explicitly require the use of rapidly growing cells in such high volumes as to preclude the inclusion of complex tissues or primary cell cultures in their implementation. Due to this requirement, HTS are designed and performed in cell lines almost exclusively [62]. Therefore, where previously we have examined the effect of IS treatment on primary slice cultures *in vitro*, to suit the goals of the present study, we specifically chose to examine the effects of our *in vitro* penumbral mimic in two types of brain cell lines to determine cell death phenotypes and molecular profiles mediated by IS. Our primary finding that IS induced a mixed phenotype of apoptosis and autophagy in neurons and astrocytes indicates this model is suitable for the development of HTS to elucidate potential neuroprotective agents against autophagy and apoptosis pathways in the ischemic penumbra. Nonetheless, putative hits generated from any such HTS will require validation in more complex biological systems, first in primary slice culture, and then *in vivo*. Despite these limitations, such a high throughput approach promises to provide far more rapid elucidation and development of potential therapeutic targets than presently utilized low-throughput approaches.

## Supporting Information

Figure S1 IS-induces mitochondrial fission. IS and oligomycin A treated mitochondria were smaller, while STS-treated apoptotic mitochondria were longer and more numerous than controls; both groups had reduced cristae density. (**A&B**) Summary of fold-changes in AIF (A) and cytochrome C (B) release from Fig. 3B. (**C)** Summary of mitochondrial morphology-related parameters from Fig. 3A. (**D**) Summary of cristae morphology-related parameters from Fig. 3A. Data are mean ± SEM. Asterisks (*) indicate significant difference from untreated controls (*p*<0.05).
(TIFF)

Figure S2 IS upregulates autophagy-related genes in neurons and astrocytes. (**A–C**) Summary of fold-changes in PI3K (A), mTOR (B), and atg7 (C) mRNA expression following 6 hrs treatment as indicated. (**D**) Sample Western blots of autophagy-related light chain 3 I and II isoform protein expression from cells treated as indicated for 6 hrs. (**E**) Summary of fold-change in protein expression ratio of LC3II/LC3I from (D) normalized to α–actin expression in the same sample. Data are mean ± SEM from 3 replicates for each experiment. Asterisks (*) indicate significant difference from untreated controls (*p*<0.05).
(TIFF)

## References

[1] Branston NM, Symon L, Crockard HA, Pasztor E (1974) Relationship between the cortical evoked potential and local cortical blood flow following acute middle cerebral artery occlusion in the baboon. Exp Neurol 45: 195–208. 4424726 10.1016/0014-4886(74)90112-5

[2] Olsen TS, Larsen B, Herning M, Skriver EB, Lassen NA (1983) Blood flow and vascular reactivity in collaterally perfused brain tissue. Evidence of an ischemic penumbra in patients with acute stroke. Stroke 14: 332–341. 6658900 10.1161/01.str.14.3.332

[3] Lo EH (2008) A new penumbra: transitioning from injury into repair after stroke. Nat Med 14: 497–500. 18463660 10.1038/nm1735

[4] Candelario-Jalil E (2009) Injury and repair mechanisms in ischemic stroke: considerations for the development of novel neurotherapeutics. Curr Opin Investig Drugs 10: 644–654. 19579170

[5] Broughton BR, Reutens DC, Sobey CG (2009) Apoptotic mechanisms after cerebral ischemia. Stroke 40: e331–339. 19182083 10.1161/STROKEAHA.108.531632

[6] Rami A, Kogel D (2008) Apoptosis meets autophagy-like cell death in the ischemic penumbra: Two sides of the same coin? Autophagy 4: 422–426. 18319639 10.4161/auto.5778

[7] Sharp FR, Lu A, Tang Y, Millhorn DE (2000) Multiple molecular penumbras after focal cerebral ischemia. J Cereb Blood Flow Metab 20: 1011–1032. 10908035 10.1097/00004647-200007000-00001

[8] Galluzzi L, Maiuri MC, Vitale I, Zischka H, Castedo M, et al (2007) Cell death modalities: classification and pathophysiological implications. Cell Death Differ 14: 1237–1243. 17431418 10.1038/sj.cdd.4402148

[9] Hansen AJ (1985) Effect of anoxia on ion distribution in the brain. Physiol Rev 65: 101–148. 3880896 10.1152/physrev.1985.65.1.101

[10] Benchoua A, Guegan C, Couriaud C, Hosseini H, Sampaio N, et al (2001) Specific caspase pathways are activated in the two stages of cerebral infarction. J Neurosci 21: 7127–7134. 11549723 10.1523/JNEUROSCI.21-18-07127.2001 PMC6762989

[11] Yao H, Shu Y, Wang J, Brinkman BC, Haddad GG (2007) Factors influencing cell fate in the infarct rim. J Neurochem 100: 1224–1233. 17217421 10.1111/j.1471-4159.2006.04299.x

[12] Anderson RE, Tan WK, Martin HS, Meyer FB (1999) Effects of glucose and PaO2 modulation on cortical intracellular acidosis, NADH redox state, and infarction in the ischemic penumbra. Stroke 30: 160–170. 9880405 10.1161/01.str.30.1.160

[13] Zauner A, Daugherty WP, Bullock MR, Warner DS (2002) Brain oxygenation and energy metabolism: part I-biological function and pathophysiology. Neurosurgery 51: 289–301; discussion 302. 12182767

[14] Rami A (2008) Upregulation of Beclin 1 in the ischemic penumbra. Autophagy 4: 227–229. 18075295 10.4161/auto.5339

[15] Hossmann KA (2008) Cerebral ischemia: models, methods and outcomes. Neuropharmacology 55: 257–270. 18222496 10.1016/j.neuropharm.2007.12.004

[16] Anderson RE, Tan WK, Meyer FB (1999) Brain acidosis, cerebral blood flow, capillary bed density, and mitochondrial function in the ischemic penumbra. J Stroke Cerebrovasc Dis 8: 368–379. 17895190 10.1016/s1052-3057(99)80044-5

[17] Yao H, Sun X, Gu X, Wang J, Haddad GG (2007) Cell death in an ischemic infarct rim model. J Neurochem 103: 1644–1653. 17727626 10.1111/j.1471-4159.2007.04879.x

[18] Yao H, Felfly H, Wang J, Zhou D, Haddad GG (2009) DIDS protects against neuronal injury by blocking Toll-like receptor 2 activated-mechanisms. J Neurochem 108: 835–846. 19077053 10.1111/j.1471-4159.2008.05838.x PMC2782475

[19] Lehnardt S, Massillon L, Follett P, Jensen FE, Ratan R, et al (2003) Activation of innate immunity in the CNS triggers neurodegeneration through a Toll-like receptor 4-dependent pathway. Proc Natl Acad Sci U S A 100: 8514–8519. 12824464 10.1073/pnas.1432609100 PMC166260

[20] Pamenter ME, Ali SS, Tang Q, Finley JC, Gu XQ, et al (2012) An in vitro ischemic penumbral mimic perfusate increases NADPH oxidase-mediated superoxide production in cultured hippocampal neurons. Brain Res 1452: 165–172. 22459046 10.1016/j.brainres.2012.03.004 PMC3326196

[21] Pamenter ME, Ryu J, Hua ST, Perkins GA, Mendiola VL, et al (2012) DIDS Prevents Ischemic Membrane Degradation in Cultured Hippocampal Neurons by Inhibiting Matrix Metalloproteinase Release. PLoS One 7: e43995. 22937143 10.1371/journal.pone.0043995 PMC3427179

[22] Karaman MW, Herrgard S, Treiber DK, Gallant P, Atteridge CE, et al (2008) A quantitative analysis of kinase inhibitor selectivity. Nat Biotechnol 26: 127–132. 18183025 10.1038/nbt1358

[23] Chae HJ, Kang JS, Byun JO, Han KS, Kim DU, et al (2000) Molecular mechanism of staurosporine-induced apoptosis in osteoblasts. Pharmacol Res 42: 373–381. 10987998 10.1006/phrs.2000.0700

[24] Roberton AM, Holloway CT, Knight IG, Beechey RB (1968) A comparison of the effects of NN′-dicyclohexylcarbodi-imide, oligomycin A and aurovertin on enrgy-linked reactions in mitochondria and submitochondrial particles. Biochem J 108: 445–456. 4299126 10.1042/bj1080445 PMC1198830

[25] Morimoto BH, Koshland DE Jr (1990) Induction and expression of long- and short-term neurosecretory potentiation in a neural cell line. Neuron 5: 875–880. 1980069 10.1016/0896-6273(90)90347-i

[26] Leist M, Single B, Castoldi AF, Kuhnle S, Nicotera P (1997) Intracellular adenosine triphosphate (ATP) concentration: a switch in the decision between apoptosis and necrosis. J Exp Med 185: 1481–1486. 9126928 10.1084/jem.185.8.1481 PMC2196283

[27] Akanda N, Elinder F (2006) Biophysical properties of the apoptosis-inducing plasma membrane voltage-dependent anion channel. Biophys J 90: 4405–4417. 16581845 10.1529/biophysj.105.080028 PMC1471872

[28] Dagda RK, Gusdon AM, Pien I, Strack S, Green S, et al (2011) Mitochondrially localized PKA reverses mitochondrial pathology and dysfunction in a cellular model of Parkinson’s disease. Cell Death Differ 18: 1914–1923. 21637291 10.1038/cdd.2011.74 PMC3177020

[29] Ju WK, Kim KY, Lindsey JD, Angert M, Patel A, et al (2009) Elevated hydrostatic pressure triggers release of OPA1 and cytochrome C, and induces apoptotic cell death in differentiated RGC-5 cells. Mol Vis 15: 120–134. 19169378 PMC2629709

[30] Boulares AH, Yakovlev AG, Ivanova V, Stoica BA, Wang G, et al (1999) Role of poly(ADP-ribose) polymerase (PARP) cleavage in apoptosis. Caspase 3-resistant PARP mutant increases rates of apoptosis in transfected cells. J Biol Chem 274: 22932–22940. 10438458 10.1074/jbc.274.33.22932

[31] Chaitanya GV, Steven AJ, Babu PP (2010) PARP-1 cleavage fragments: signatures of cell-death proteases in neurodegeneration. Cell Commun Signal 8: 31. 21176168 10.1186/1478-811X-8-31 PMC3022541

[32] Fadok VA, Voelker DR, Campbell PA, Cohen JJ, Bratton DL, et al (1992) Exposure of phosphatidylserine on the surface of apoptotic lymphocytes triggers specific recognition and removal by macrophages. J Immunol 148: 2207–2216. 1545126

[33] Koopman G, Reutelingsperger CP, Kuijten GA, Keehnen RM, Pals ST, et al (1994) Annexin V for flow cytometric detection of phosphatidylserine expression on B cells undergoing apoptosis. Blood 84: 1415–1420. 8068938

[34] Karbowski M, Youle RJ (2003) Dynamics of mitochondrial morphology in healthy cells and during apoptosis. Cell Death Differ 10: 870–880. 12867994 10.1038/sj.cdd.4401260

[35] Banasiak KJ, Cronin T, Haddad GG (1999) bcl-2 prolongs neuronal survival during hypoxia-induced apoptosis. Brain Res Mol Brain Res 72: 214–225. 10529480 10.1016/s0169-328x(99)00189-8

[36] Matsushita K, Matsuyama T, Kitagawa K, Matsumoto M, Yanagihara T, et al (1998) Alterations of Bcl-2 family proteins precede cytoskeletal proteolysis in the penumbra, but not in infarct centres following focal cerebral ischemia in mice. Neuroscience 83: 439–448. 9460752 10.1016/s0306-4522(97)00391-6

[37] Tanida I, Ueno T, Kominami E (2008) LC3 and Autophagy. Methods Mol Biol 445: 77–88. 18425443 10.1007/978-1-59745-157-4_4

[38] Kroemer G, Jaattela M (2005) Lysosomes and autophagy in cell death control. Nat Rev Cancer 5: 886–897. 16239905 10.1038/nrc1738

[39] Puyal J, Vaslin A, Mottier V, Clarke PG (2009) Postischemic treatment of neonatal cerebral ischemia should target autophagy. Ann Neurol 66: 378–389. 19551849 10.1002/ana.21714

[40] Barone FC (2009) Ischemic stroke intervention requires mixed cellular protection of the penumbra. Curr Opin Investig Drugs 10: 220–223. 19333878

[41] Lazebnik YA, Takahashi A, Moir RD, Goldman RD, Poirier GG, et al (1995) Studies of the lamin proteinase reveal multiple parallel biochemical pathways during apoptotic execution. Proc Natl Acad Sci U S A 92: 9042–9046. 7568069 10.1073/pnas.92.20.9042 PMC40920

[42] Hattori K, Kajimura M, Hishiki T, Nakanishi T, Kubo A, et al (2010) Paradoxical ATP elevation in ischemic penumbra revealed by quantitative imaging mass spectrometry. Antioxid Redox Signal 13: 1157–1167. 20486758 10.1089/ars.2010.3290 PMC2956403

[43] Kalinowska M, Garncarz W, Pietrowska M, Garrard WT, Widlak P (2005) Regulation of the human apoptotic DNase/RNase endonuclease G: involvement of Hsp70 and ATP. Apoptosis 10: 821–830. 16133872 10.1007/s10495-005-0410-9

[44] Wang J, Wei Q, Wang CY, Hill WD, Hess DC, et al (2004) Minocycline up-regulates Bcl-2 and protects against cell death in mitochondria. J Biol Chem 279: 19948–19954. 15004018 10.1074/jbc.M313629200

[45] Morin D, Pires F, Plin C, Tillement JP (2004) Role of the permeability transition pore in cytochrome C release from mitochondria during ischemia-reperfusion in rat liver. Biochem Pharmacol 68: 2065–2073. 15476677 10.1016/j.bcp.2004.07.006

[46] Repici M, Centeno C, Tomasi S, Forloni G, Bonny C, et al (2007) Time-course of c-Jun N-terminal kinase activation after cerebral ischemia and effect of D-JNKI1 on c-Jun and caspase-3 activation. Neuroscience 150: 40–49. 17900813 10.1016/j.neuroscience.2007.08.021

[47] Maiuri MC, Zalckvar E, Kimchi A, Kroemer G (2007) Self-eating and self-killing: crosstalk between autophagy and apoptosis. Nat Rev Mol Cell Biol 8: 741–752. 17717517 10.1038/nrm2239

[48] Yao H, Takasawa R, Fukuda K, Shiokawa D, Sadanaga-Akiyoshi F, et al (2001) DNA fragmentation in ischemic core and penumbra in focal cerebral ischemia in rats. Brain Res Mol Brain Res 91: 112–118. 11457498 10.1016/s0169-328x(01)00135-8

[49] Culmsee C, Zhu C, Landshamer S, Becattini B, Wagner E, et al (2005) Apoptosis-inducing factor triggered by poly(ADP-ribose) polymerase and Bid mediates neuronal cell death after oxygen-glucose deprivation and focal cerebral ischemia. J Neurosci 25: 10262–10272. 16267234 10.1523/JNEUROSCI.2818-05.2005 PMC6725791

[50] Weinstein PR, Hong S, Sharp FR (2004) Molecular identification of the ischemic penumbra. Stroke 35: 2666–2670. 15486332 10.1161/01.STR.0000144052.10644.ed

[51] Krupinski J, Lopez E, Marti E, Ferrer I (2000) Expression of caspases and their substrates in the rat model of focal cerebral ischemia. Neurobiol Dis 7: 332–342. 10964605 10.1006/nbdi.2000.0310

[52] Rami A, Langhagen A, Steiger S (2008) Focal cerebral ischemia induces upregulation of Beclin 1 and autophagy-like cell death. Neurobiol Dis 29: 132–141. 17936001 10.1016/j.nbd.2007.08.005

[53] Ferrer I, Friguls B, Dalfo E, Justicia C, Planas AM (2003) Caspase-dependent and caspase-independent signalling of apoptosis in the penumbra following middle cerebral artery occlusion in the adult rat. Neuropathol Appl Neurobiol 29: 472–481. 14507339 10.1046/j.1365-2990.2003.00485.x

[54] Kroemer G, El-Deiry WS, Golstein P, Peter ME, Vaux D, et al (2005) Classification of cell death: recommendations of the Nomenclature Committee on Cell Death. Cell Death Differ 12 Suppl 21463–1467. 16247491 10.1038/sj.cdd.4401724

[55] Graham SH, Chen J (2001) Programmed cell death in cerebral ischemia. J Cereb Blood Flow Metab 21: 99–109. 11176275 10.1097/00004647-200102000-00001

[56] Fink SL, Cookson BT (2005) Apoptosis, pyroptosis, and necrosis: mechanistic description of dead and dying eukaryotic cells. Infect Immun 73: 1907–1916. 15784530 10.1128/IAI.73.4.1907-1916.2005 PMC1087413

[57] Yakovlev AG, Faden AI (2004) Mechanisms of neural cell death: implications for development of neuroprotective treatment strategies. NeuroRx 1: 5–16. 15717003 10.1602/neurorx.1.1.5 PMC534908

[58] Gelot A, Villapol S, Billette de Villemeur T, Renolleau S, Charriaut-Marlangue C (2009) Astrocytic demise in the developing rat and human brain after hypoxic-ischemic damage. Dev Neurosci 31: 459–470. 19672074 10.1159/000232564

[59] Xu L, Sapolsky RM, Giffard RG (2001) Differential sensitivity of murine astrocytes and neurons from different brain regions to injury. Exp Neurol 169: 416–424. 11358455 10.1006/exnr.2001.7678

[60] del Zoppo GJ (2010) The neurovascular unit, matrix proteases, and innate inflammation. Ann N Y Acad Sci 1207: 46–49. 20955425 10.1111/j.1749-6632.2010.05760.x PMC4547552

[61] del Zoppo GJ, Frankowski H, Gu YH, Osada T, Kanazawa M, et al (2012) Microglial cell activation is a source of metalloproteinase generation during hemorrhagic transformation. J Cereb Blood Flow Metab 32: 919–932. 22354151 10.1038/jcbfm.2012.11 PMC3345906

[62] Zhang JH, Chung TD, Oldenburg KR (1999) A Simple Statistical Parameter for Use in Evaluation and Validation of High Throughput Screening Assays. J Biomol Screen 4: 67–73. 10838414 10.1177/108705719900400206

