# Supplementary figures and images for: Autophagy and Apoptosis Are Differentially Induced in Neurons and Astrocytes Treated with an In Vitro Mimic of the Ischemic Penumbra
Source: PLoS One. 2012 Dec 12;7(12):e51469. doi: 10.1371/journal.pone.0051469 (PMC3520810; doi:10.1371/journal.pone.0051469)

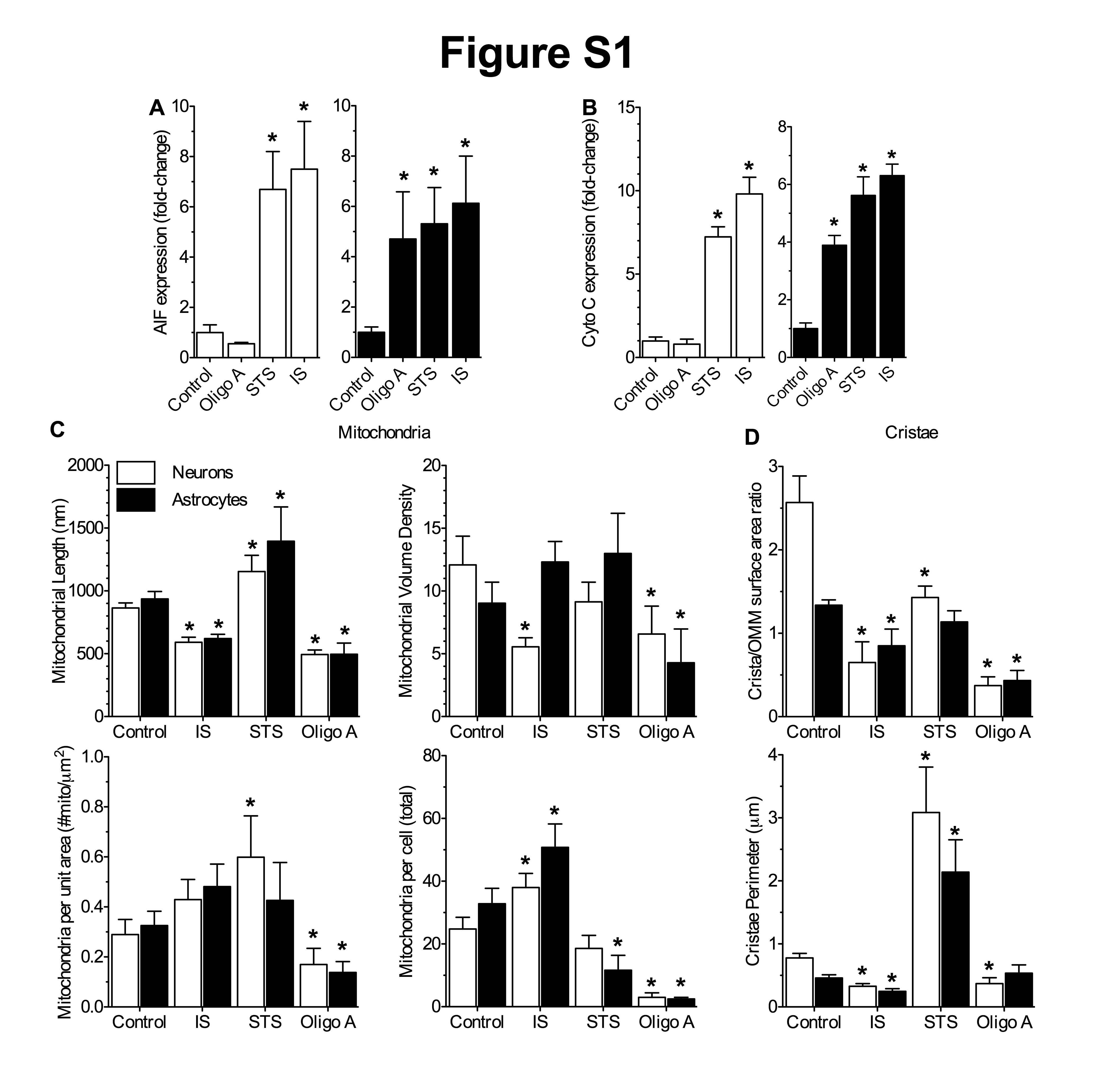

Supplement: Figure S1 — IS-induces mitochondrial fission. IS and oligomycin A treated mitochondria were smaller, while STS-treated apoptotic mitochondria were longer and more numerous than controls; both groups had reduced cristae density. (A&B) Summary of fold-changes in AIF (A) and cytochrome C (B) release from Fig. 3B. (C) Summary of mitochondrial morphology-related parameters from Fig. 3A. (D) Summary of cristae morphology-related parameters from Fig. 3A. Data are mean ± SEM. Asterisks (*) indicate significant difference from untreated controls (p<0.05). (TIFF) [file pone.0051469.s001.tiff]

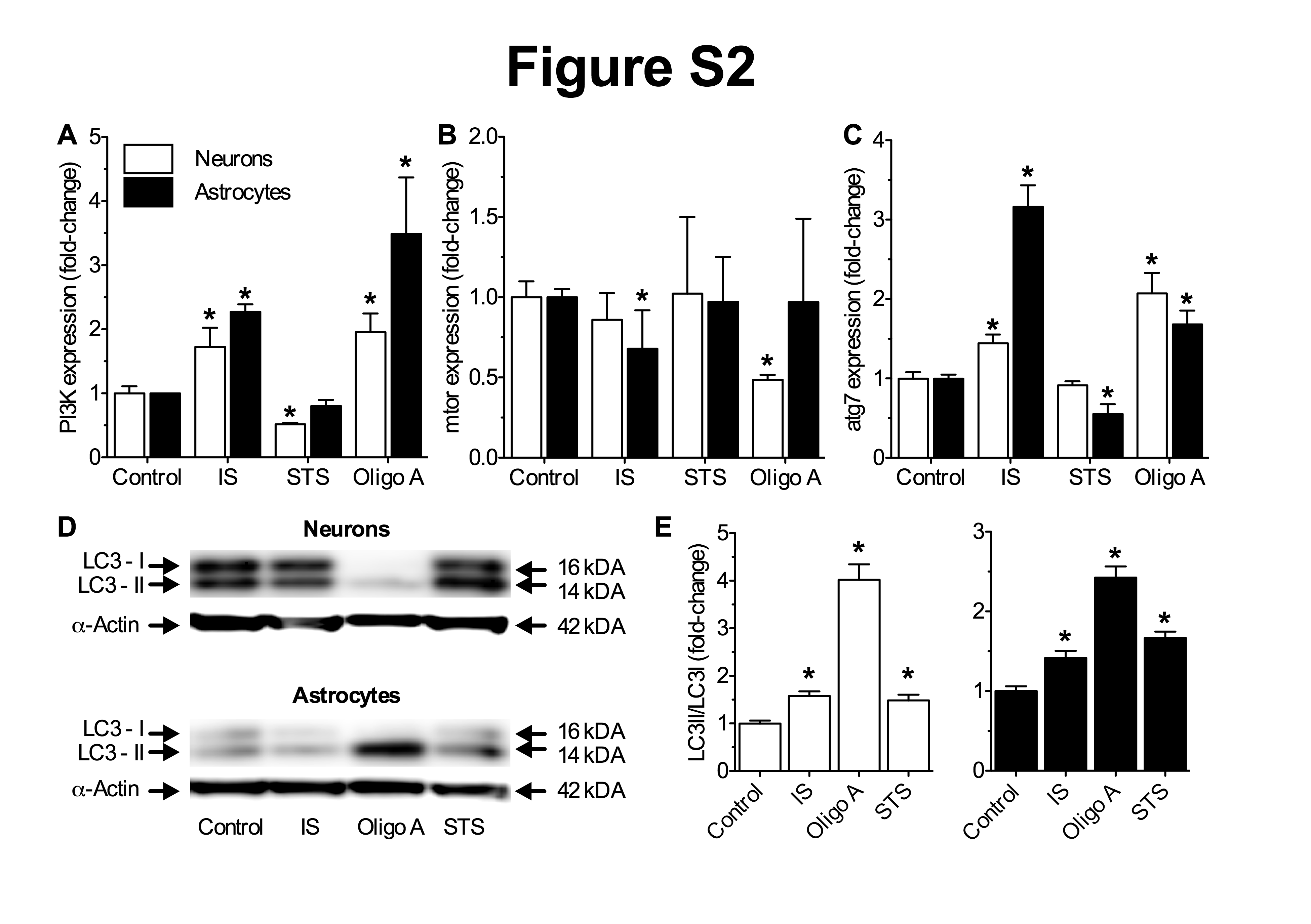

Supplement: Figure S2 — IS upregulates autophagy-related genes in neurons and astrocytes. (A–C) Summary of fold-changes in PI3K (A), mTOR (B), and atg7 (C) mRNA expression following 6 hrs treatment as indicated. (D) Sample Western blots of autophagy-related light chain 3 I and II isoform protein expression from cells treated as indicated for 6 hrs. (E) Summary of fold-change in protein expression ratio of LC3II/LC3I from (D) normalized to α–actin expression in the same sample. Data are mean ± SEM from 3 replicates for each experiment. Asterisks (*) indicate significant difference from untreated controls (p<0.05). (TIFF) [file pone.0051469.s002.tiff]
